# miRNA-197 and miRNA-223 Predict Cardiovascular Death in a Cohort of Patients with Symptomatic Coronary Artery Disease

**DOI:** 10.1371/journal.pone.0145930

**Published:** 2015-12-31

**Authors:** Christian Schulte, Simon Molz, Sebastian Appelbaum, Mahir Karakas, Francisco Ojeda, Denise M. Lau, Tim Hartmann, Karl J. Lackner, Dirk Westermann, Renate B. Schnabel, Stefan Blankenberg, Tanja Zeller

**Affiliations:** 1 Department of General and Interventional Cardiology, University Heart Center Hamburg Eppendorf, Hamburg, Germany; 2 German Center for Cardiovascular Research (DZHK), Partner site Hamburg/Luebeck/Kiel, Hamburg, Germany; 3 Institute of Clinical Chemistry and Laboratory Medicine, University Medical Center Mainz, Mainz, Germany; 4 German Center for Cardiovascular Research (DZHK), Partner site Rhein-Main, Mainz, Germany; Gustave Roussy, FRANCE

## Abstract

**Background:**

Circulating microRNAs (miRNAs) have been described as potential diagnostic biomarkers in cardiovascular disease and in particular, coronary artery disease (CAD). Few studies were undertaken to perform analyses with regard to risk stratification of future cardiovascular events. miR-126, miR-197 and miR-223 are involved in endovascular inflammation and platelet activation and have been described as biomarkers in the diagnosis of CAD. They were identified in a prospective study in relation to future myocardial infarction.

**Objectives:**

The aim of our study was to further evaluate the prognostic value of these miRNAs in a large prospective cohort of patients with documented CAD.

**Methods:**

Levels of miR-126, miR-197 and miR-223 were evaluated in serum samples of 873 CAD patients with respect to the endpoint cardiovascular death. miRNA quantification was performed using real time polymerase chain reaction (RT-qPCR).

**Results:**

The median follow-up period was 4 years (IQR 2.78–5.04). The median age of all patients was 64 years (IQR 57–69) with 80.2% males. 38.9% of the patients presented with acute coronary syndrome (ACS), 61.1% were diagnosed with stable angina pectoris (SAP). Elevated levels of miRNA-197 and miRNA-223 reliably predicted future cardiovascular death in the overall group (miRNA-197: hazard ratio (HR) 1.77 per one standard deviation (SD) increase (95% confidence interval (CI) 1.20; 2.60), p = 0.004, C-index 0.78; miRNA-223: HR 2.23 per one SD increase (1.20; 4.14), p = 0.011, C-index 0.80). In ACS patients the prognostic power of both miRNAs was even higher (miRNA-197: HR 2.24 per one SD increase (1.25; 4.01), p = 0.006, C-index 0.89); miRA-223: HR 4.94 per one SD increase (1.42; 17.20), p = 0.012, C-index 0.89).

**Conclusion:**

Serum-derived circulating miRNA-197 and miRNA-223 were identified as predictors for cardiovascular death in a large patient cohort with CAD. These results reinforce the assumption that circulating miRNAs are promising biomarkers with prognostic value with respect to future cardiovascular events.

## Introduction

In cardiovascular pathologies circulating miRNAs have been described as disease-specific biomarkers [1–7] and various animal models and clinical studies have proven miRNAs suitable for diagnostic purposes in coronary artery disease (CAD) and myocardial infarction (MI) [2, 8–14]. Numerous miRNAs were successfully evaluated as circulating biomarkers in the diagnosis of CAD and MI and their functional role in cardiovascular primary prevention has been suggested [15].

Endothelial expressed circulating miR-126 has formerly been reported to be dysregulated in stable CAD [2, 16], in symptomatic stable angina pectoris (SAP) [17], and in acute MI [18, 19]. Elevated serum levels of miR-223 were reported in patients with acute MI [20] and miR-223 has been shown to be a moderator of endovascular high-density lipoprotein associated anti-inflammatory effects [21]. Platelet-derived miR-197 levels are associated with thrombocyte activation and were found altered in patients receiving anti-platelet therapy [22], while miR-197 was dysregulated in patients with type 2 diabetes mellitus [23] indicating a potential role of this miRNA in CAD disease progression. Only a few studies have been performed based on large clinical cohorts and little is known about the added value of miRNAs as prospective biomarkers for risk stratification of future cardiovascular events. One study evaluated miRNAs regarding their applicability in primary prevention [24]. miR-126, miR-197 and miR-223 were found to be associated with an elevated risk for future myocardial infarction [24].

The aim of the present study was to gain further knowledge about the prognostic value of miR-126, miR-197 and miR-223 in a large cohort of 873 consecutive patients with documented CAD with respect to the endpoint cardiovascular death. miR-197 and miR-223 were identified as predictors of cardiovascular death.

## Methods

### Study sample

The Athero*Gene* study is a prospective cohort study of consecutive patients with manifest CAD. Patients enrolled in the study had been admitted to the Department of Medicine II of the University Clinic Mainz as well as the Department of Medicine of the German Federal Armed Forces Central Hospital Koblenz between June 1999 and March 2000 for diagnostic coronary angiography performed due to symptoms of suspected CAD [25]. At least one stenosis of 30% or more present in a major coronary artery was diagnosed in all patients included in the study. Patients were excluded if there was evidence of hemodynamically significant valvular heart disease, a history of coronary artery bypass graft surgery, percutaneous transluminal coronary angioplasty or trauma within the previous month, known cardiomyopathy, cancer, febrile conditions or use of oral anticoagulant therapy. Patients presenting with sepsis, cardiopulmonary resuscitation, or pulmonary embolism within the last four weeks were also excluded. In AtheroGene 3,800 patients were initially recruited. After subjects with missing laboratory measurements, missing information on the cause of death, or incomplete information on the clinical presentation of CAD as well as subjects with missing samples or low sample volume were ruled out, a subcohort of 1,112 of these patients was included in the study. Finally, after selection of complete cases 873 patients were included in the miRNA quantification analyses (**S1 Fig**). There were no relevant differences in baseline characteristics between the complete cases cohort and the available cases cohort (**S1 Table**).

The study was performed according to the Declaration of Helsinki. The protocol of the study was approved by the ethics committee of the Johannes Gutenberg-University Mainz and the Physicians’ chamber of the State Rheinland Pfalz (Germany). Participation in the study was voluntary and all subjects gave their written informed consent before they entered the study. The primary endpoint of this study was the endpoint cardiovascular death.

### Data collection

All subjects filled in a standardized questionnaire providing socio-demographic information and medical history. Further data were taken from hospital charts. Acute myocardial infarction was defined as either STEMI with significant ST-segment elevation in at least two contiguous leads or non-STEMI according to clinical presentation and positive in-house troponin concentrations. The diagnosis of unstable angina pectoris was made according to Braunwald.

Information on adverse events was obtained from the patients by mailed standardized questionnaires as well as telephone interviews after discharge and verified by information from the primary care physicians by standardized questionnaire until a mean follow-up time of four years. Information about the cause of death was obtained from hospital or general practitioner charts. When information from medical charts was insufficient, death certificates were checked. Deaths from cardiovascular causes, besides fatal MI and fatal stroke, also included heart failure as a consequence of MI and ventricular arrhythmia. The cause of death was then coded according to the International Classification of Diseases.

Serum samples were collected on admission before coronary angiography under standardized conditions, centrifuged, aliquoted and stored at -80°C until measurements. Further details of the study have been published before [26].

### RNA isolation

Laboratory personnel were blinded to study endpoints by using pseudonymized sample identification numbers. Reproducibility was performed by measurement of duplicates of some of the samples. Methods have been described in [14].

Circulating cell-free total RNA was isolated from frozen serum samples using Trizol reagent (Invitrogen) and purified by the miRNeasy kit (Qiagen) as described before [14]. Briefly, 3 volumes of Trizol were mixed with 1 volume of serum samples and incubated for 5 min at room temperature. Chloroform was added, and after 5 minutes at room temperature the mixture was centrifuged at 14,000 g and 4°C for 15 min. The upper aqueous phase was transferred to a fresh reagent tube and 1.5 volumes ethanol were added. Purification of RNA was performed with miRNeasy according to the manufactures´ recommendations. RNA was eluted in 30μl RNase-free H_2_O. For normalization, serum samples were supplemented with 10nM *Caenorhabditis elegans* miR-39 (cel-miR-39) after addition of Trizol.

### Detection of circulating miRNA

RNA was reverse transcribed to cDNA using the TaqMan MicroRNA Reverse Transcription Kit and the Megaplex™ RT primers (Megaplex RT Primer Pool A) (both Applied Biosystems). Real-time PCR amplification of miRNAs miR-126, miR-223, miR-197 and C. elegans miR-39 was performed on an Applied Biosystems 7900 HT system using miRNA-specific TaqMan assays (Applied Biosystems). PCR runs included one cycle of 50°C for 2 min and 95°C for 20 sec followed by 40 cycles of 95°C for 1 sec and 60°C for 20 sec. Quantitative analysis was performed using the SDS software v2.4 (Applied Biosystems). The assays and specific miRNA sequences that were used are listed in **S1 File**. miRNA detection was defined by the normalized cycle threshold (Ct) values.

### Statistical analysis

Ct values were normalized to cel-miR-39 and miRNA expression is presented as deltaCt = Ct[miRNA]-Ct[cel-miRNA-39]. If miRNA Ct ≥ 40, the value was considered as undetermined and was set to deltaCt = 40. Analyzes were restricted to individuals with complete information on age, sex, follow-up variables and miRNA levels (n = 873). Continuous variables are presented as median plus 25^th^ percentile and 75^th^ percentile.

Spearman-rank correlations were computed to analyze statistical dependence between miRNAs, ACS-SAP stratified Cox regression models, adjusted for age, sex and further cardiovascular risk factors were used to determine the association of each miRNA to cardiovascular death. Principal component analysis (PCA), a variable reduction technique, was used to reduce the number of covariates used in the models. The aim of principal component analysis is to reduce the dimension of a dataset, while retaining as much as possible of the variation in the dataset. PCA of the variables that are intended to be used for adjusting the Cox models is used to reduce the number of variables in case of small number of events. The proportional hazard assumption and the log linearity for continuous variables were tested, and no evidence of violation was found.

In order to enhance the interpretation of the hazard ratios positive deltaCt values were transferred into negative deltaCt values and used as independent variables, which reverses the inverse relationship between Ct values and miRNA concentration.

In order to assess the prognostic value of any combination of the three miRNAs Cox regression of all possible models (single miRNAs and different miRNA combinations) were computed. All models were based on the same individuals with available data for all three miRNAs and endpoint variables (All: N = 873, 18 events; ACS: N = 340, 8 events; SAP: N = 533, 10 events). Subsequently, these models were compared by Akaike information criterion (AIC) as described before [27]. AIC negatively rates an increase of parameters so that the favored model is indicated by lower AIC values. This approach allows an optimized weighing between effort and benefit with regard to the number of parameters included in an analysis. Ten-fold cross validation was used to internally validate the C-indices of all Cox models computed, controlling for the overoptimism of estimating performance measures using the same dataset the model was computed with.

A two-sided p-value <0.05 was considered as statistical significant. All analyses were performed using R software, Version 3.1.2 [28].

## Results

### Study characteristics

873 patients with 38.9% (n = 340) cases of acute coronary syndrome (ACS) and 61.1% (n = 533) cases of stable angina pectoris (SAP) were included in the study.

Cardiovascular death was observed in 2.1% (n = 18) of the patients over a median follow-up time of 4 years (IQR 2.78–5.04). The study characteristics of all study participants are provided in **Table 1**. Distribution of patients with future cardiovascular death as primary end point was not significantly different between the ACS and SAP groups (**Table 1**).

**Table 1:** Characteristics of subjects of the AtheroGene study.

	All (n = 873)	ACS (n = 340)	SAP (n = 533)	p-value
**Male gender (%)**	700 (80.2)	276 (81.2)	424 (79.5)	0.62
**Age**	64 (57, 69)	63 (56, 68)	64 (59, 70)	0.001
**BMI (kg/m** ^**2**^ **)**	27 (25, 30)	27 (24.7, 29.4)	27.1 (25, 30.1)	0.08
**Ever smoker (%)**	535 (61.3)	225 (66.2)	310 (58.2)	0.021
**History of diabetes (%)**	186 (21.3)	56 (16.5)	130 (24.4)	0.007
**Hypertension (%)**	690 (79)	241 (70.9)	449 (84.2)	<0.001
**History of MI at baseline (%)**	308 (35.3)	92 (27.1)	216 (40.5)	<0.001
**Dyslipidemia (%)**	631 (72.3)	215 (63.2)	416 (78)	<0.001
**Primary end point cardiovascular death (%)**	18 (2.1)	8 (2.4)	10 (1.9)	0.81

Continuous variables are presented as median (25^th^; 75^th^ percentile). For discrete variables the absolute (relative) frequencies are given. BMI = body mass index, MI = myocardial infarction.

Baseline levels of all three miRNAs were higher in patients with future cardiovascular death compared with event-free subjects (**S2 Table)**.

**Fig 1:**
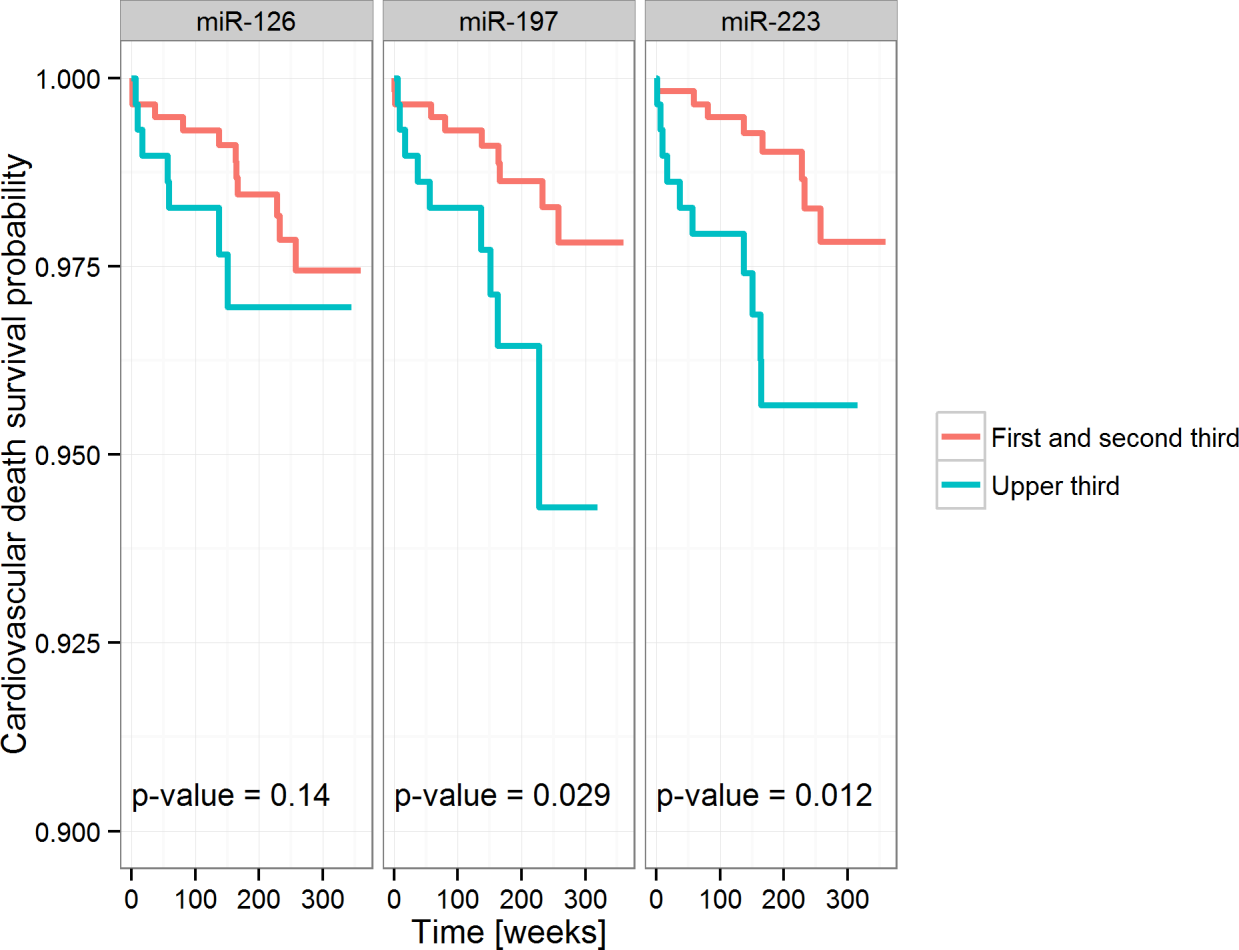
Kaplan Meier Plots for each analyzed miRNA. miRNA levels are dichotomized using their second tertile. miRNA levels are presented in minus ΔCT values so that larger values correspond to high miRNA concentrations. The p-values given describe the log-rank test.

**Fig 1** depicts survival curves according to dichotomized miRNA levels. For miR-197 and miR-223 the analysis showed significantly reduced survival in patients with higher circulating miRNA levels.

### Association of miRNA levels with cardiovascular outcome

Correlations between miR-126, miR-197 and miR-223 were moderate to high (**Table 2**).

**Table 2:** Correlations of the miRNAs.

	miR-126	miR-197	miR-223
**miR-126**	1.00		
**miR-197**	0.69	1.00	
**miR-223**	0.56	0.60	1.00

Cox regression analysis adjusted for age and gender revealed relevant prognostic power of miR-197 and miR-223 with respect to the primary end point cardiovascular death in the overall group (miRNA-197: HR 1.77 per one SD increase (95% CI 1.20; 2.60), p = 0.004, C-index 0.78; miRNA-223: HR 2.23 per one SD increase (1.20; 4.14), p = 0.011, C-index 0.80), (**Table 3**). Subgroup analysis for ACS patients revealed a stronger association between elevated levels of miR-197 and miR-223 and future cardiovascular death (miRNA-197: HR 2.24 per one SD increase (1.25; 4.01), p = 0.006, C-index 0.89); miRNA-223: HR 4.94 per one SD increase (1.42; 17.20), p = 0.012, C-index 0.89) (**Table 3**). None of the analyzed miRNAs showed prognostic power for SAP patients. Levels of miR-126 showed no significant prognostic value, neither in the overall group nor for the ACS or the SAP group (**Table 3**). The prognostic value of miR-197 and miR-223 remained unchanged when Cox regression models were additionally adjusted for conventional cardiovascular risk factors (Body Mass Index, diabetes mellitus, hypertension, history of myocardial infarction, hyperlipidemia, ever smoker) (**S3 Table**). After adjustment for cardiovascular risk factors miR-126 showed prognostic value with respect to incident cardiovascular death (**S3 Table**). After application of PCA the prognostic value remained unchanged (**S3 Table)**.

**Table 3:** Cox regression analysis for the prognostic power of miR-126, miR-197 and miR-223 with respect to the endpoint cardiovascular death, adjusted for age and gender including hazard ratios of age and sex.

Group	miRNA	HR per 1 SD	2.5% CI	97.5% CI	Event rate	P-value	C-index
**All**							
	miR-126	1.25	0.69	2.26	2.1%	0.447	0.69
	Age	3.23	1.61	6.5		<0.001	
	Male	0.76	0.52	1.12		0.17	
	miR-197	1.77	1.20	2.60	2.1%	0.004	0.78
	Age	2.91	1.47	5.78		0.002	
	Male	0.78	0.53	1.15		0.21	
	miR-223	2.23	1.2	4.14	2.1%	0.011	0.80
	Age	3.0	1.51	5.97		0.002	
	Male	0.79	0.54	1.16		0.22	
**ACS**							
	miR-126	0.94	0.44	2.03	2.4%	0.880	0.80
	Age	2.87	0.99	8.27		0.052	
	Male	0.59	0.34	1.02		0.06	
	miR-197	2.24	1.25	4.01	2.4%	0.006	0.89
	Age	2.38	0.84	6.75		0.10	
	Male	0.63	0.36	1.11		0.11	
	miR-223	4.94	1.42	17.20	2.4%	0.012	0.89
	Age	2.67	0.98	7.25		0.055	
	Male	0.61	0.35	1.05		0.075	
**SAP**							
	miR-126	1.54	0.70	3.37	1.9%	0.280	0.73
	Age	3.47	1.39	8.66		0.008	
	Male	0.95	0.54	1.66		0.86	
	miR-197	1.48	0.85	2.57	1.9%	0.166	0.74
	Age	3.37	1.35	8.39		0.009	
	Male	0.95	0.55	1.66		0.86	
	miR-223	1.48	0.76	2.89	1.9%	0.250	0.75
	Age	3.54	1.41	8.92		0.007	
	Male	0.98	0.56	1.72		0.954	

ACS: acute coronary syndrome, CI: confidence interval, HR: Hazard ratio, SAP: stable angina pectoris, SD: standard deviation

### Combination of miRNAs

To test whether a combination of miR-126, miR-197 and miR-223 increases the prognostic power for future cardiovascular death AIC values and C-indexes were calculated for any combination of the three miRNAs. AIC values and C-indexes showed no differences between single miRNAs and combinations of miRNAs in all groups tested (**Table 4**). Thus, there was no additional benefit of analyzing a miRNA combination compared with single miR-197 or miR-223.

**Table 4:** Akaike Information Criterion analysis of miR-126, miR-197 and miR-223 as well as combinations of these miRNAs.

Group	miRNA	AIC	C-index
**All**			
	**miR-126**	196.26	0.66
	**miR-197**	189.81	0.76
	**miR-223**	190.28	0.79
	**miR-126 + miR-197**	191.53	0.75
	**miR-126 + miR-223**	192.27	0.72
	**miR-197 + miR-223**	190.29	0.77
	**miR-126 + miR-197 + miR-223**	191.68	0.76
**ACS**			
	**miR-126**	84.48	0.53
	**miR-197**	78.06	0.80
	**miR-223**	77.69	0.84
	**miR-126 + miR-197**	79.30	0.68
	**miR-126 + miR-223**	79.12	0.70
	**miR-197 + miR-223**	78.01	0.78
	**miR-126 + miR-197 + miR-223**	78.83	0.72
**SAP**			
	**miR-126**	115.46	0.70
	**miR-197**	115.00	0.71
	**miR-223**	115.30	0.73
	**miR-126 + miR-197**	116.90	0.71
	**miR-126 + miR-223**	116.92	0.72
	**miR-197 + miR-223**	116.72	0.71
	**miR-126 + miR-197 + miR-223**	118.66	0.71

Cox regression of possible miRNA combinations were compared by AIC, which negatively rates an increase of parameters so that the favored model is indicated by lower AIC values. ACS: acute coronary syndrome, AIC: Akaike Information Criterion, SAP: stable angina pectoris

## Discussion

The present study aimed to evaluate the prognostic value of circulating miR-126, miR-197, and miR-223 in a secondary prevention cohort with respect to future cardiovascular events. Circulating levels of miR-197 and miR-223 were identified as prognostic biomarkers of future cardiovascular death.

### Diagnostic and prognostic value of miRNAs in CAD and ACS

The discovery of circulating miRNAs as promising novel biomarkers in the field of cardiovascular disease has created great expectations. One promising application of circulating miRNAs is their possible use as prognostic biomarkers for CAD [2] and risk stratification [29]. The results of our study extend the applicability of circulating miRNAs towards secondary prevention in a cohort of patients with angiographically diagnosed CAD.

Serum levels of miR-223 were identified as significantly elevated in patients with atherosclerosis[30] suggesting a role in CAD. From a pathophysiological point of view miR-223 is involved in the regulation of multiple inflammatory genes[31, 32] as well as cholesterol homeostasis[33]. miR-197 is strongly associated with platelet activation [22] and diabetes mellitus [23].

Subgroup analysis of ACS patients revealed an even higher association of baseline miRNA-197 and -223 levels and future cardiovascular death. These results are clinically relevant as they show the potential to improve individual secondary prevention strategies for ACS patients with a high risk of future cardiovascular complications and death. miR-223 is known to be associated with activation of platelets [22] and all three analyzed miRNAs have been shown to be highly expressed in thrombocytes [24]. Activated platelets correlate to increased mortality in patients with ACS even months and years after the initial event. This is less frequently observed in patients with SAP, where obstructive CAD and plaques within coronary arteries are less common. This is supported by our data showing differing results between ACS and SAP patients with respect to prognostic power of miRNAs for future cardiovascular death.

Generally, the combinatory testing of multiple biomarkers performs better compared with single marker approaches and represents a necessity in evaluation of new biomarkers. In this study, AIC analysis revealed no benefit of multiple testing. This might be an expression of the correlation of the analyzed miRNAs among each other.

So far, the prognostic value of miRNAs has been evaluated in one large-scale, primary prevention trial only. In 820 apparently healthy subjects, three circulating miRNAs around endothelium-enriched miR-126 were associated with MI in the general population suggesting these miRNAs as biomarkers in primary prevention of CAD [24]. As a limitation, all three miRNAs identified failed in crude, in age- and sex-adjusted, and in conventional risk factor-adjusted analyses. The reported miRNAs did not reach statistical significance until adjustment was broadly extended to various other measures like fibrinogen and waist-to-hip ratio. More importantly, in a subanalysis none of the 3 miRNAs were prognostic for early (years 1995 to 2000) AND late (years 2000 to 2005) events, thereby strongly questioning the reliability of the results. Contrary to that report, miR-126 did not show a statistically significant association with future cardiovascular death in age and gender adjusted Cox regression in our study. These differences may be attributed to the different end points used in our study and by Zampetaki et al.–MI and cardiovascular death. Comparability of the results may also be compromised by the use of different biomaterial (serum vs. plasma) [34], normalization methods [35], and patient characteristics. Additionally, while miR-126 was formerly found to be down regulated in ACS [19], Zampetaki et al. reported elevated levels in patients with future MI, indicating discrepancies between miRNA utilization as prognostic biomarkers on the one hand and diagnostic biomarkers on the other. Nevertheless, our data for the first time identified circulating miRNAs as predictors for cardiovascular death in a large-scale clinical trial with CAD patients.

### Limitations

In our study the number of cardiovascular death end point is rather small. This may limit the statistical ability to detect small effects and contains the risk of statistically overfitting the results, especially with respect to the subgroups ACS and SAP. Thus, future studies are needed to evaluate our observation.

### Clinical implications

From a clinical point of view our data suggest a promising applicability of miR-197 and miR-223 in cardiovascular risk stratification of CAD patients. Complementary use of miRNAs as circulating biomarkers might further enable physicians to predict the risk for future cardiovascular events, improve secondary prevention and lower cardiovascular mortality.

## Conclusions

In conclusion, we were able to identify miR-197 and miR-223 as predictors for cardiovascular death in a large cohort of documented CAD patients. Our results suggest the potential use of circulating miRNAs as prognostic biomarkers in secondary prevention in the field of cardiovascular disease. Future large-scale studies are needed to further evaluate these findings.

## Supporting Information

S1 Fig Work flow of the patient selection process in the AtheroGene study. The primary patient cohort comprised of 3,800 patients. After subjects with missing laboratory measurements, missing information on the cause of death, or incomplete information on the clinical presentation of CAD were ruled out, 3,423 patients were included in the available cases cohort. 2,311 pts. were excluded due to incomplete serum samples. After 239 patients were excluded for missing miRNA data the complete cases cohort consisted of 873 patients. [1] Zampetaki et al. JACC, 2012; [2] Fichtlscherer et al. Circulation research, 2010; [3] Tabet et al. Nature communications, 2014; [4] Zampetaki et al. Circulation research, 2010
(TIFF)

S1 File miRNA Assays. (DOCX)

S1 Table Baseline characteristics of available patients in AtheroGene and complete cases analysed in this study. Continuous variables are presented as median (25^th^; 75^th^ percentile). For discrete variables the absolute (relative) frequencies are given. CVRF = cardiovascular risk factors
(DOCX)

S2 Table Distribution of miRNAs according to outcome. miRNA levels are presented as ΔCT values. Variables are shown as normalized median values.
(DOCX)

S3 Table Association of circulating miRNAs with cardiovascular death during follow-up, adjusted for age, gender and additional cardiovascular risk factors, stratified for ACS and SAP. Cardiovascular risk factors: Body Mass Index, diabetes mellitus, hypertension, history of myocardial infarction, hyperlipidemia, ever smoker. PCA = Principal component analysis; PCA of the variables that are intended to be used for adjusting the Cox models was used to reduce the number of variables in case of small number of events. Cox regression analyses are shown with and without application of PCA (“Yes” and “No”).
(DOCX)
