# Supplementary figures and images for: miRNA-197 and miRNA-223 Predict Cardiovascular Death in a Cohort of Patients with Symptomatic Coronary Artery Disease
Source: PLoS One. 2015 Dec 31;10(12):e0145930. doi: 10.1371/journal.pone.0145930 (PMC4699820; doi:10.1371/journal.pone.0145930)

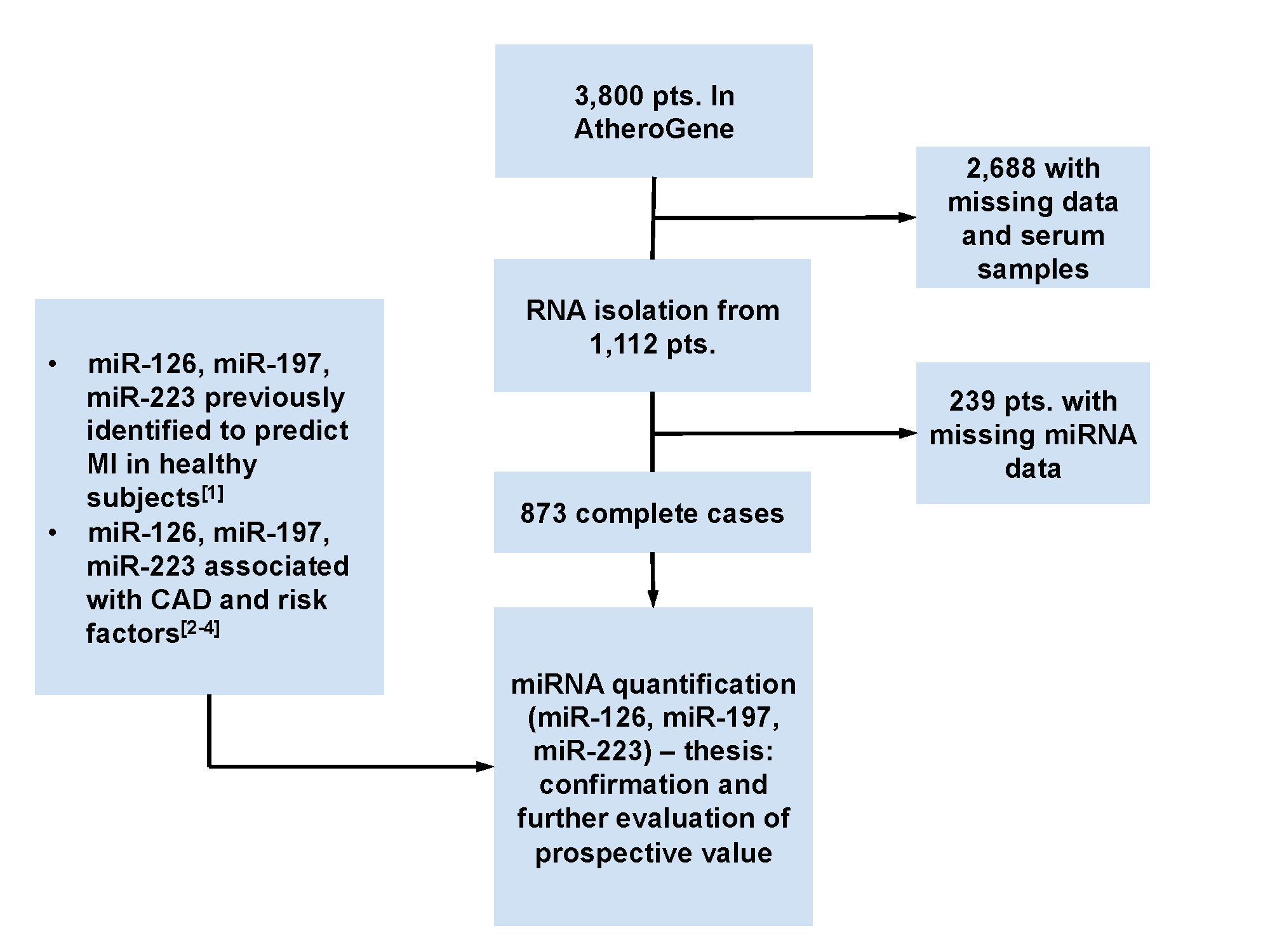

Supplement: S1 Fig — The primary patient cohort comprised of 3,800 patients. After subjects with missing laboratory measurements, missing information on the cause of death, or incomplete information on the clinical presentation of CAD were ruled out, 3,423 patients were included in the available cases cohort. 2,311 pts. were excluded due to incomplete serum samples. After 239 patients were excluded for missing miRNA data the complete cases cohort consisted of 873 patients. [1] Zampetaki et al. JACC, 2012; [2] Fichtlscherer et al. Circulation research, 2010; [3] Tabet et al. Nature communications, 2014; [4] Zampetaki et al. Circulation research, 2010 (TIFF) [file pone.0145930.s001.tiff]
