# Supplementary material for: miRNA-197 and miRNA-223 Predict Cardiovascular Death in a Cohort of Patients with Symptomatic Coronary Artery Disease
Source: PLoS One. 2015 Dec 31;10(12):e0145930. doi: 10.1371/journal.pone.0145930 (PMC4699820; doi:10.1371/journal.pone.0145930)
Supplement: S1 File — (DOCX) [file pone.0145930.s002.docx]

**miRNA-39**

cel-miR-39, Life Technology; Assay 000200, mature miRNA sequence: UCACCGGGUGUAAAUCAGCUUG

**miRNA-126**

hsa-miR-126, Life Technology; Assay 002228, mature miRNA sequence: UCGUACCGUGAGUAAUAAUGCG

**miRNA-197**

hsa-miR-197, Life Technology; Assay 000497, mature miRNA sequence: UUCACCACCUUCUCCACCCAGC

**miRNA-223**

hsa-miR-223, Life Technology; Assay 002295, mature miRNA sequence: UGUCAGUUUGUCAAAUACCCCA
