# Supplementary material for: miRNA-197 and miRNA-223 Predict Cardiovascular Death in a Cohort of Patients with Symptomatic Coronary Artery Disease
Source: PLoS One. 2015 Dec 31;10(12):e0145930. doi: 10.1371/journal.pone.0145930 (PMC4699820; doi:10.1371/journal.pone.0145930)
Supplement: S1 Table — Continuous variables are presented as median (25th; 75th percentile). For discrete variables the absolute (relative) frequencies are given. CVRF = cardiovascular risk factors (DOCX) [file pone.0145930.s003.docx]

|  | **All (n=3423)** | **Complete cases (n=873)** |
| --- | --- | --- |
| **Male gender (%)** | 2,573 (75.2) | 700 (80.2) |
| **Age** | 63 (55, 69.8) | 64 (57, 69) |
| **Cardiovascular Risk Factors** |  |  |
| **BMI (kg/m²)** | 27 (24.9, 29.7) | 27 (25, 30) |
| **Ever smoker (%)** | 2,135 (62.4) | 535 (61.3) |
| **History of diabetes (%)** | 739 (21.6) | 186 (21.3) |
| **Hypertension (%)** | 2,547 (74.4) | 690 (79) |
| **History of MI at baseline (%)** | 1,401 (40.9) | 308 (35.3) |
| **Dyslipidemia (%)** | 2,318 (67.7) | 631 (72.3) |
