# Supplementary material for: miRNA-197 and miRNA-223 Predict Cardiovascular Death in a Cohort of Patients with Symptomatic Coronary Artery Disease
Source: PLoS One. 2015 Dec 31;10(12):e0145930. doi: 10.1371/journal.pone.0145930 (PMC4699820; doi:10.1371/journal.pone.0145930)
Supplement: S2 Table — miRNA levels are presented as ΔCT values. Variables are shown as normalized median values. (DOCX) [file pone.0145930.s004.docx]

| **miRNA** | **All** | **No Event** | **Event** |
| --- | --- | --- | --- |
| **miR-126 ΔCT (IQR)** | 9.34 (6.85, 11.02) | 9.34 (6.86, 11.0) | 8.63 (4.15, 11.19) |
| **miR-197 ΔCT (IQR)** | 12.61 (10.34, 14.07) | 12.62 (10.38, 14.07) | 11.07 (7.45, 13.55) |
| **miR-223 ΔCT (IQR)** | 11.02 (7.86, 13.06) | 11.04 (7.98, 13.1) | 8.04 (4.23, 11.51) |
