# Supplementary material for: miRNA-197 and miRNA-223 Predict Cardiovascular Death in a Cohort of Patients with Symptomatic Coronary Artery Disease
Source: PLoS One. 2015 Dec 31;10(12):e0145930. doi: 10.1371/journal.pone.0145930 (PMC4699820; doi:10.1371/journal.pone.0145930)
Supplement: S3 Table — Cardiovascular risk factors: Body Mass Index, diabetes mellitus, hypertension, history of myocardial infarction, hyperlipidemia, ever smoker. PCA = Principal component analysis; PCA of the variables that are intended to be used for adjusting the Cox models was used to reduce the number of variables in case of small number of events. Cox regression analyses are shown with and without application of PCA (“Yes” and “No”). (DOCX) [file pone.0145930.s005.docx]

| **Group** | **miRNA** | **PCA** | **HR per 1 SD** | **2.5% CI** | **97.5% CI** | **P-value** | **C-index** |
| --- | --- | --- | --- | --- | --- | --- | --- |
| **All** |  |  |  |  |  |  |  |
|  | **miR-126** | No | 2.34 | 1.16 | 4.73 | 0.018 | 0.78 |
|  |  | Yes | 2.38 | 1.25 | 4.54 | 0.0087 | 0.75 |
|  | **miR-197** | No | 1.79 | 1.17 | 2.74 | 0.0077 | 0.79 |
|  |  | Yes | 1.84 | 1.24 | 2.72 | 0.0024 | 0.76 |
|  | **miR-223** | No | 2.59 | 1.29 | 5.20 | 0.0073 | 0.81 |
|  |  | Yes | 2.39 | 1.28 | 4.43 | 0.006 | 0.79 |
| **ACS** |  |  |  |  |  |  |  |
|  | **miR-126** | No | 4.04 | 1.07 | 15.24 | 0.039 | 0.95 |
|  |  | Yes | 5.15 | 1.61 | 16.52 | 0.0058 | 0.88 |
|  | **miR-197** | No | 2.05 | 1.01 | 4.17 | 0.047 | 0.95 |
|  |  | Yes | 2.47 | 1.34 | 4.54 | 0.0036 | 0.9 |
|  | **miR-223** | No | 6.66 | 1.39 | 31.93 | 0.018 | 0.94 |
|  |  | Yes | 6.61 | 1.79 | 24.42 | 0.0046 | 0.9 |
| **SAP** |  |  |  |  |  |  |  |
|  | **miR-126** | No | 1.56 | 0.67 | 3.62 | 0.30 | 0.77 |
|  |  | Yes | 1.74 | 0.81 | 3.73 | 0.16 | 0.68 |
|  | **miR-197** | No | 1.53 | 0.83 | 2.84 | 0.18 | 0.79 |
|  |  | Yes | 1.63 | 0.95 | 2.79 | 0.077 | 0.68 |
|  | **miR-223** | No | 1.68 | 0.79 | 3.57 | 0.18 | 0.8 |
|  |  | Yes | 1.64 | 0.84 | 3.17 | 0.14 | 0.74 |
